# Poverty from fetal life onward and child brain morphology

**DOI:** 10.1038/s41598-023-28120-2

**Published:** 2023-01-23

**Authors:** Yuna Koyama, Andrea P. Cortes Hidalgo, Rebecca E. Lacey, Tonya White, Pauline W. Jansen, Takeo Fujiwara, Henning Tiemeier

**Affiliations:** 1 Department of Global Health Promotion, Tokyo Medical and Dental University (TMDU), Tokyo, Japan; 2 Department of Child and Adolescent Psychiatry/Psychology, Erasmus MC University Medical Center Rotterdam, Rotterdam, The Netherlands; 3 Generation R Study Group, Erasmus MC University Medical Center Rotterdam, Rotterdam, The Netherlands; 4 Department of Epidemiology and Public Health, University College London, London, UK; 5 Department of Psychology, Education and Child Studies, Erasmus University Rotterdam, Rotterdam, The Netherlands; 6 Department of Social and Behavioral Sciences, Harvard T.H. Chan School of Public Health, Boston, MA USA

**Keywords:** Neuroscience, Risk factors, Socioeconomic scenarios, Medical research, Epidemiology, Paediatric research

## Abstract

Poverty is a risk factor for impaired child development, an association possibly mediated by brain morphology. Previous studies lacked prospective poverty assessments during pregnancy and did not stratify by majority/minority status. We investigated the association of household poverty from fetal life forward with brain morphological differences at age 10 years, in 2166 mother–child dyads. Overall, the results showed no associations between any poverty exposure early in life and brain volumes. However, there was the evidence of timing effects: children exposed to poverty in utero had smaller amygdala volumes (B =  − 0.18, 95%CI − 0.30; − 0.07, *p*_FDR-adjusted_ = 0.009). There were also differences in associations by majority/minority status (cerebral white matter: p for interaction = 0.04). Dutch children exposed to childhood poverty showed smaller cerebral white matter volumes than their control (B =  − 0.26, 95%CI − 0.45; − 0.06, *p*_FDR-adjusted_ = 0.035). This association was not observed in the minority population (B =  − 0.05, 95%CI − 0.23; 0.12, *p*_FDR-adjusted_ = 0.542). The smaller cerebral white matter volume mediated the association between childhood poverty and poorer school performance in Dutch children. Our findings point to the importance of poverty exposure in the fetal period and suggest different mechanisms and vulnerabilities across majority/minority groups.

## Introduction

Poverty is a well-known determinant of numerous dimensions of child development^1,2^. In addition to poor physical development, impaired cognitive functions and socioemotional development consistently occur more often in children exposed to poverty^3^. According to the ecosocial theory of disease distribution, poverty can become biologically embedded and this can underlie population health inequality including child developmental disparities^4^. Child brain development has been examined as a neurobiological factor possibly mediating these associations^5,6^. Poverty is related to brain developmental disadvantages due to deprivation of cognitive stimulation, inadequate nutrition, exposure to environmental toxins and psychological stress^7^, which perpetuate structural inequalities in society^8^. Most studies reported positive associations between income and total gray and white matter volumes^5,9,10^, indicating that poverty and structural deprivation have a global impact on brain development, possibly as part of stunted growth. Other researches on child exposure to low income ^5,6,10,11^ focused on regions of interest, in particular the hippocampus and amygdala. These studies are conducted against the background that these subcortical structures, which are rich in cortisol receptors, are more sensitive to stress^12^. Studies examining poverty and the hippocampal and amygdala volumes yielded mixed findings, with some reporting smaller volumes of the hippocampus ^5,10,11^ and amygdala ^6,10,13^ and others no association with the hippocampus ^6^ and amygdala^5,14^. These inconsistent findings might be due to small sample sizes ^5,6,9–11,13,15^. In addition, only few studies were conducted outside of the US^6,11,16^. The US and Western European countries are different in terms of welfare policy, ^17^ the level of inequality ^18^ and poverty rate^19^; hence the impact of poverty may differ and studies in non-US countries are important to explore generalizability of results.

A few studies examined whether brain morphology mediated the association between income and cognitive functions^5,14^. In a large cross-sectional study of 389 participants aged 4–22 years, those from low-income household scored lower on IQ tests than those from high- or middle-income households, and approximately 20% of this association could be explained by smaller volumes of the frontal and temporal lobes^5^. Similarly, in individuals aged between 3 and 20 years, whole-brain surface area partially accounted for the association between household income and executive functions^14^. These studies were cross-sectional. Mediation models based on such cross-sectional measures cannot be interpreted temporally, and thus a cautious causal inference is not possible^20^. Prospective studies are needed to evaluate whether important functional consequences of low household family income, such as less optimal offspring cognitive function, are explained by differences in brain morphology.

Brain development starts rapidly prenatally, and although it continues beyond adolescence, the volumes of many structures already approach their maximum volume 2 years after birth^21^. The different developmental trajectories of each region ^21,22^ could underlie a differential impact of prenatal and postnatal poverty. Also, critical brain developmental processes, such as the neuronal migration and gyrification, occur primarily during the prenatal period^23^. Thus, exposure to adverse conditions in fetal life, such as famine, could have long-term implications^23^. Children institutionalized from birth showed smaller hippocampal volumes, which was followed by catch-up only among those placed in higher quality care before 18 months old^12,24^. These reports support a critical period of brain development from fetal period to infancy. However, little is known about the role of timing in the association between poverty and brain morphology since most studies in childhood or adolescence were cross-sectional.

Importantly, minority status and poverty co-occur in many societies^25^. Minority populations often experience institutional and cultural discrimination (e.g. residential segregation and negative stereotypes), which can lead to differences in socioeconomic status^26^. Some scholars argue that racial disparities in health largely reflect differences in socioeconomic status between majority and minority populations, yet racial health disparities often remain after taking socioeconomic status into account^26^. Others argue that minority status and poverty interact in the relation with poor health outcomes^27^. Among migrants, poverty status may be tied to inequity and discrimination, and the resulting stress that can impact child development may be greater than in majority groups^28^. A previous study from our current cohort showed associations between exposure to prenatal stress and offspring IQ only in ethnic minorities^29^. Therefore, examining whether there are differences in the association between poverty and brain morphology by majority and minority status is critical but, to the best of our knowledge, has not been done.

In the current study, we investigated the association between exposure to poverty, defined as living in a family with household income below the national low-income threshold, and child brain morphology. In line with previous findings of an association between poverty and global brain metrics^5,9,10,13,15^, we hypothesized that poverty would be associated with smaller total brain, cortical gray matter, and cerebral white matter volumes. Next, we examined the association between timing of exposure to poverty and child brain morphology. The timing of exposure was categorized into prenatal period and early childhood (postnatal period) within the first five years of life. We hypothesized that prenatal exposure to poverty is more strongly associated with differences in brain morphology than postnatal exposure in line with the fetal origins hypothesis ^30^ and the ecosocial theory^4^. The prenatal period is a particularly vulnerable stage of brain development, with ongoing neurogenesis and neuronal migration, synaptogenesis, and myelination in the second and third trimester^31^. Any environmental stimuli in this stage will likely be influential^32^. Also, we hypothesized that poverty may be differentially associated with these structural brain differences in majority and minority groups due to immigration patterns that explain selection effects (e.g. the self-selection of migrants by personality characteristics and motivation), the lack of social support in the host country, less financial assets, and, importantly, the unique experience of discrimination by minorities. We had no a priori hypothesis on specific regions that would be differentially associated with such cumulative effects and experiences in minority or majority groups only. Further, in a post-hoc analysis, we examined whether any association of exposure to poverty with offspring brain morphology might underlie differences in cognitive functions as captured by school performance at a later age.

## Results

**Figure 1:**
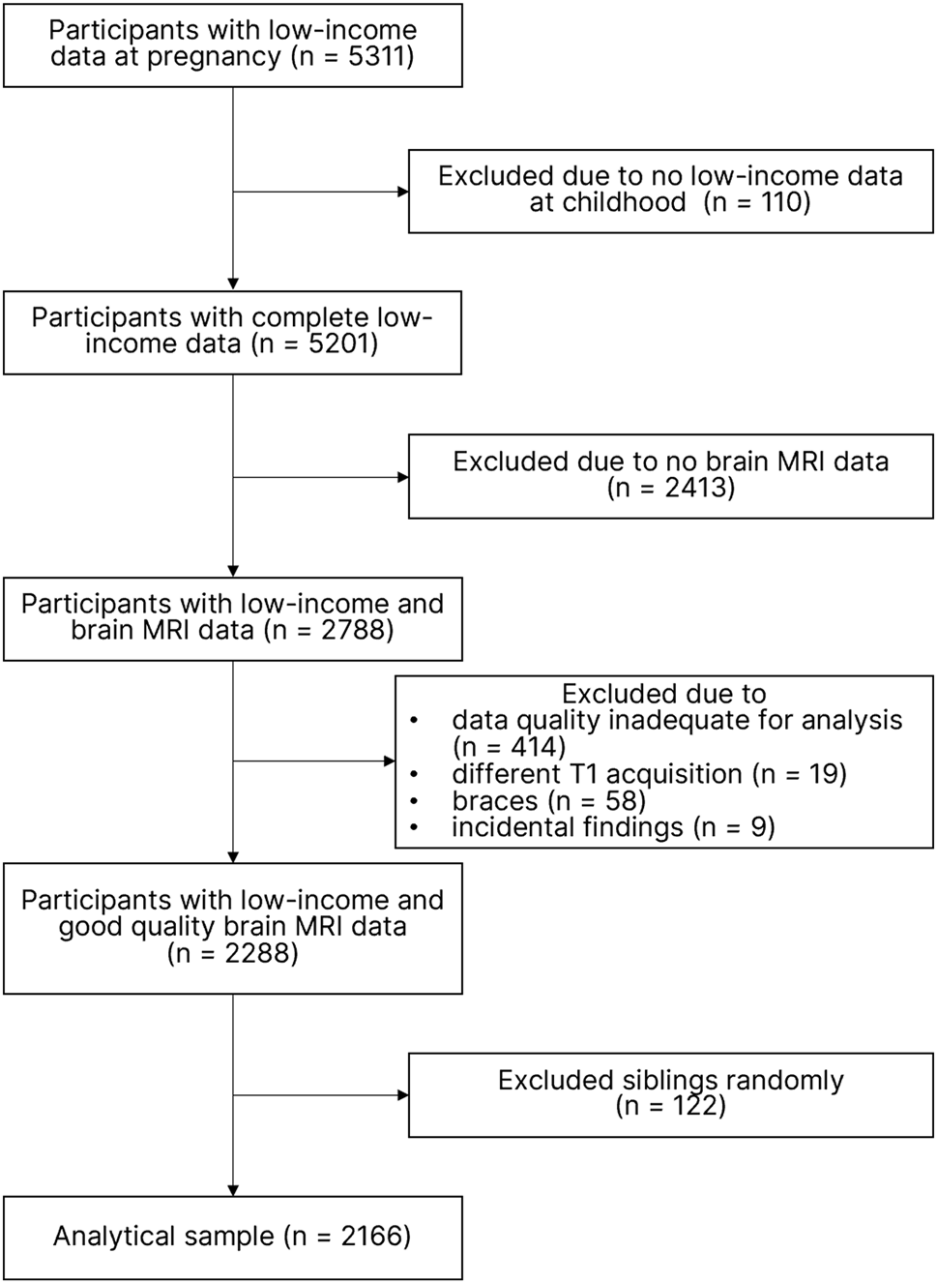
Sampling flow chart.

**Figure 2:**
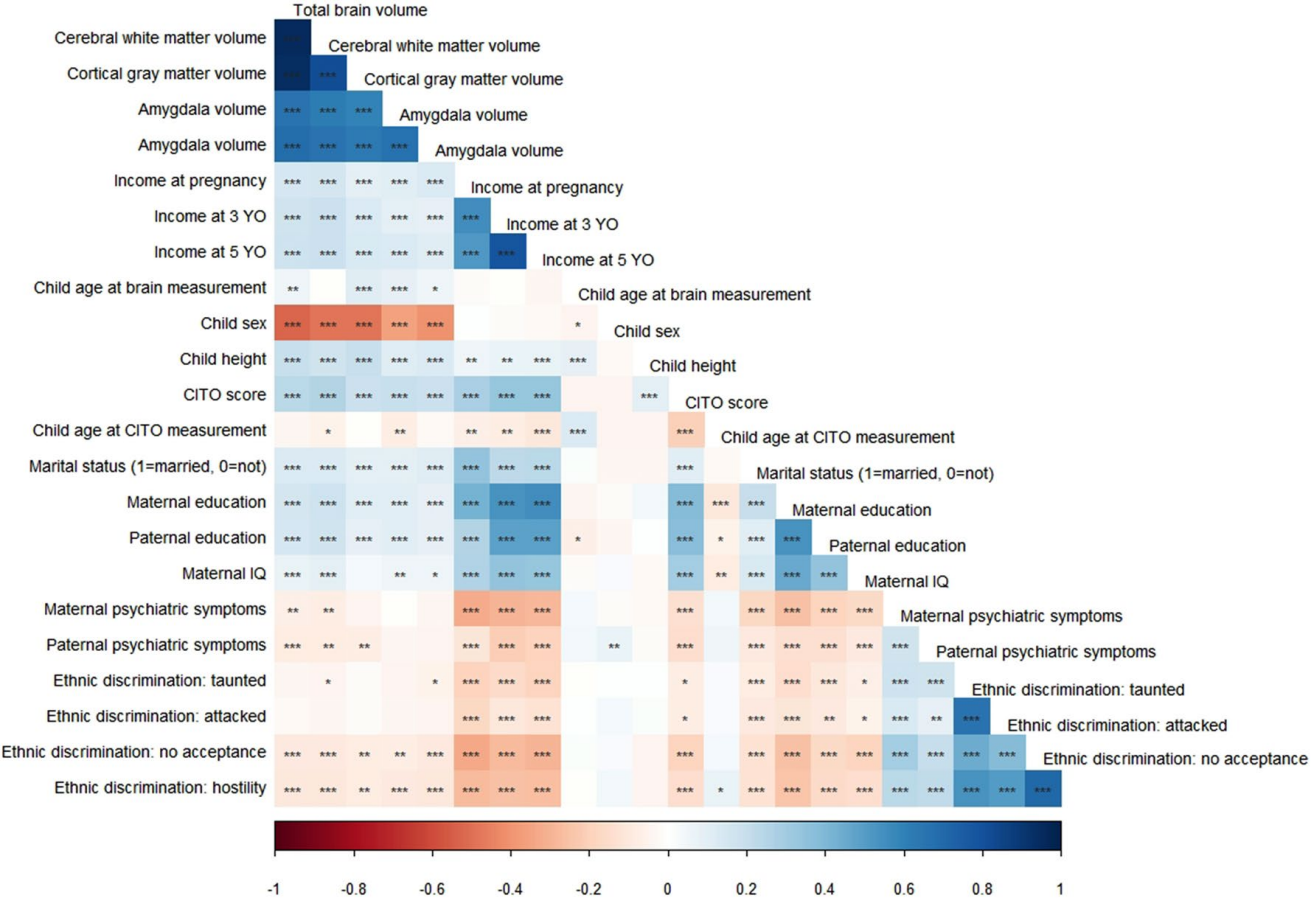
Correlation matrix of child brain morphology, household income, and child and familial demographic characteristics. The color grading gives the correlation strengths. Complete cases were analyzed. * indicates *p* < 0.05, ** indicates *p* < 0.01, *** indicates *p* < 0.001.

Data from the Generation R Study, a prospective population-based birth cohort in Rotterdam, the Netherlands, was analyzed^33^. In total, 5311 pregnant women provided data on standardized household income in pregnancy. After excluding those without data on poverty status and brain magnetic resonance imaging (MRI), and keeping one of two siblings to avoid giving more weight to certain households with multiple child participants, a total of 2166 children were left for the analytical sample (Fig. 1). Supplementary Table 1 shows the sample characteristics of those in the analytical sample and those who were lost to follow-up. Additionally, we compared maternal and child characteristic of children without brain imaging data or with poor quality brain imaging data to those of children included in the analyses (Supplementary Table 2). Most notably, children with brain MRI data came from slightly higher socioeconomic backgrounds. The correlations among variables of interests in the current study are shown in Fig. 2.

Poverty was defined by the household standardized income, calculated using family size and household income, under the national low-income threshold of the Netherlands ^e.g.^^34^. Of all children, 20.4% (n = 442) were in poverty in one or more assessment periods (Table 1): 15.1% experienced poverty in pregnancy, and 15.3% experienced poverty in childhood (when children were 3 and 5 years old). Minority was defined according to maternal national origin following definitions used by Statistics Netherlands^35^. The Netherlands do not use a race categorization but parental national origin to denote recent immigration. We collapsed these to “Dutch”, “Non-Dutch Western”, and “Non-Western”; the latter included Cape Verdean, Moroccan, Dutch Antillean, Surinamese, Turkish, other African, middle and other south American and most Asian origins. Only 115 of 1365 (8.4%) children from Dutch majority group, but 297 of 530 (56.0%) children from non-Western minority group had ever experienced poverty. The group of children that experienced poverty in childhood included 93 children of Dutch majority status (28.1%) and 220 children of non-Western minority status (66.5%). Poverty was also categorized to reflect the specific timing of poverty, i.e. exposure in pregnancy only, exposure in childhood only, and chronic exposure (Supplementary Table 3). The sample characteristics by majority and minority statuses are available in Supplementary Table 4.

**Table 1:** Sample characteristics (N = 2166).

Characteristics	Never poverty N = 1724 (79.6%)		Ever poverty N = 442		Timing of poverty exposure			
					Poverty in pregnancy, all ^a^ N = 326 (15.1%)		Poverty in childhood, all ^a^ N = 331 (15.3%)	
Child sex								
Male, N, %	843	48.9	215	48.6	158	48.5	164	49.5
Female, N, %	881	51.1	227	51.4	168	51.5	167	50.5
Child age at MRI measurement (years), mean, SD	10.1	0.6	10.2	0.6	10.2	0.5	10.1	0.5
Child school performance (CITO score), mean, SD	539.9	7.7	534.0	9.2	533.5	8.8	534.0	8.8
Maternal ethnicity								
Dutch, N, %	1250	72.5	115	26.0	57	17.5	93	28.1
Non Dutch Western, N, %	241	14.0	30	6.8	24	7.4	18	5.4
Non Western, N, %	233	13.5	297	67.2	245	75.2	220	66.5
Maternal education at pregnancy ^b^								
High, N, %	684	39.7	17	3.8	11	3.4	10	3.1
Mid-high, N, %	492	28.5	67	15.2	39	12.2	48	14.5
Mid-low, N, %	435	25.2	172	38.9	118	36.2	126	38.1
Low, N, %	113	6.6	186	42.1	158	48.5	147	44.4
Maternal IQ ^c^, mean, SD	101.0	12.5	90.3	15.0	89.1	14.9	88.8	15.0
Parental psychiatric symptoms at pregnancy								
Mother ^d^, median, IQR	0.12	0.2	0.27	0.5	0.35	0.6	0.29	0.6
Father ^e^, median, IQR	0.06	0.1	0.12	0.2	0.14	0.3	0.12	0.3

The data was combined across imputed datasets.

Non-Dutch-Western includes Indonesian, American, Asian, European, Oceanian. Non-Western includes Cape Verdean, Moroccan, Dutch Antillean, Surinamese, Turkish, African, American non-Western, Asian non-Western.

Ever poverty is a total of "poverty in pregnancy only", "poverty in childhood only" and "chronic poverty".

Abbreviations: *MRI* magnetic resonance imaging, *SD* standard deviation, *CITO* Centraal Instituut voor Test Ontwikkeling, *IQR* interquartile range.

^a^These populations are not mutually exclusive. To see the mutually exclusive sample categorization, refer to Supplementary Table 1.

^b^Missing data N = 54 (2.5%).

^c^Missing data N = 138 (6.4%).

^d^Missing data N = 233 (10.8%).

^e^Missing data N = 583 (26.9%).

### Poverty and child brain morphology by timing of poverty exposure

Child brain morphological data were collected when children were approximately at the age of 10.1 (SD: 0.6). The association between poverty experience and brain morphology was examined, adjusting for child age and sex, minority or majority status, maternal IQ, maternal educational attainment, and maternal and paternal psychiatric symptoms. We standardized the brain metrics, which means that B values per category of exposure can be compared across brain regions. We observed no association between exposure to poverty at any assessment timing and the global child brain morphology measures in the total sample (e.g. total brain volume: B =  − 0.10, 95%CI − 0.21; 0.01, *p* = 0.08), except for an association between ever being exposed to poverty in childhood and total brain volume (B =  − 0.12, 95%CI − 0.23; − 0.001, *p* = 0.05) (Table 2). As for the results on subcortical regions, which are shown in Table 3, children ever being exposed to poverty in pregnancy had smaller amygdala volumes (B =  − 0.18, 95%CI − 0.30; − 0.07, *p* < 0.01, *p*_FDR-adjusted_ = 0.009). The results for the partially adjusted models are shown in Supplementary Table 5 (for global brain metrics) and Supplementary Table 6 (for subcortical brain metrics).

**Table 2:** The association of poverty with global brain morphology (N = 2166).

	N	Total brain volume			Cortical gray matter volume			Cerebral white matter volume		
		B	95%CI	*p* value	B	95%CI	*p* value	B	95%CI	*p* value
Never poverty	1724	Ref.			Ref.			Ref.		
Ever poverty	442	− 0.10	− 0.21 to 0.01	0.08	− 0.11	− 0.22 to 0.01	0.06	− 0.09	− 0.20 to 0.03	0.14
Overall periodical effects										
No poverty in pregnancy	1840	Ref.			Ref.			Ref.		
Poverty in pregnancy, all	326	− 0.05	− 0.17 to 0.08	0.44	− 0.08	− 0.21 to 0.05	0.22	− 0.02	− 0.15 to 0.11	0.74
No poverty in childhood	1835	Ref.			Ref.			Ref.		
Poverty in childhood, all	331	− 0.12	− 0.23 to − 0.001	0.05	− 0.11	− 0.22 to 0.01	0.08	− 0.12	− 0.24 to 0.002	0.05
Specific periodical effects										
Never poverty	1724	Ref.			Ref.			Ref.		
Poverty in pregnancy only	111	− 0.03	− 0.20 to 0.15	0.76	− 0.07	− 0.25 to 0.11	0.43	0.004	− 0.17 to 0.18	0.96
Poverty in childhood only	116	− 0.14	− 0.31 to 0.02	0.08	− 0.12	− 0.28 to 0.05	0.16	− 0.16	− 0.33 to 0.01	0.06
Chronic poverty	215	− 0.10	− 0.26 to 0.05	0.17	− 0.12	− 0.27 to 0.03	0.13	− 0.08	− 0.24 to 0.07	0.29

Models adjusted for child age at brain measurement, child sex, maternal ethnicity, maternal IQ, maternal educational attainment at pregnancy, and maternal and paternal psychiatry symptoms at pregnancy.

All brain measures of outcome are standardized.

Ever poverty is a total of "poverty in pregnancy only", "poverty in childhood only" and "chronic poverty".

There is no *p* values survived the multiple comparisons corrections (four tests for ever, pragnant any and childhood any poverty and 12 tests (= four brain metrics × three timings of exposure) for pregnant, childhood and chronic poverty) with the Benjamini–Hochberg false discovery rate method.

**Table 3:** The association of poverty with subcortical regional brain morphology (N = 2166).

	N	Mean hippocampus volume			Mean amygdala volume		
		B	95%CI	*p* value	B	95%CI	*p* value
Never poverty	1724	Ref.			Ref.		
Ever poverty	442	− 0.05	− 0.15 to 0.06	0.35	− 0.11	− 0.21 to − 0.004	0.04
Overall periodical effects							
No poverty in pregnancy	1840	Ref.			Ref.		
Poverty in pregnancy, all	326	− 0.03	− 0.15 to 0.09	0.63	− 0.18	− 0.30 to − 0.07	< 0.01 **
No poverty in childhood	1835	Ref.			Ref.		
Poverty in childhood, all	331	0.00	− 0.11 to 0.11	0.96	− 0.05	− 0.16 to 0.06	0.34
Specific periodical effects							
Never poverty	1724	Ref.			Ref.		
Poverty in pregnancy only	111	− 0.12	− 0.28 to 0.04	0.15	− 0.18	− 0.35 to − 0.02	0.03
Poverty in childhood only	116	− 0.06	− 0.22 to 0.09	0.42	0.03	− 0.13 to 0.18	0.75
Chronic poverty	215	0.01	− 0.13 to 0.15	0.87	− 0.17	− 0.32 to − 0.03	0.02

Models adjusted for child age at brain measurement, child sex, maternal ethnicity, maternal IQ, maternal educational attainment at pregnancy, maternal and paternal psychiatry symptoms at pregnancy and total intracranial volume.

All brain measures of outcome are standardized.

Ever poverty is a total of "poverty in pregnancy only", "poverty in childhood only" and "chronic poverty".

** indicates adjusted *p* value < 0.01.

Adjusted *p* values are obtained by considering the multiple comparisons (four tests for ever, pragnant any and childhood any poverty and 12 tests (= four brain metrics × 3 timings of exposure) for pregnant, childhood and chronic poverty) with the Benjamini–Hochberg false discovery rate method.

As a sensitivity analysis, sex interaction with ever being exposed to poverty was examined to assess the robustness of the findings for both girls and boys. We found no interaction effect by child sex (Supplementary Table 7).

### Poverty and child brain morphology by majority and minority status

**Figure 3:**
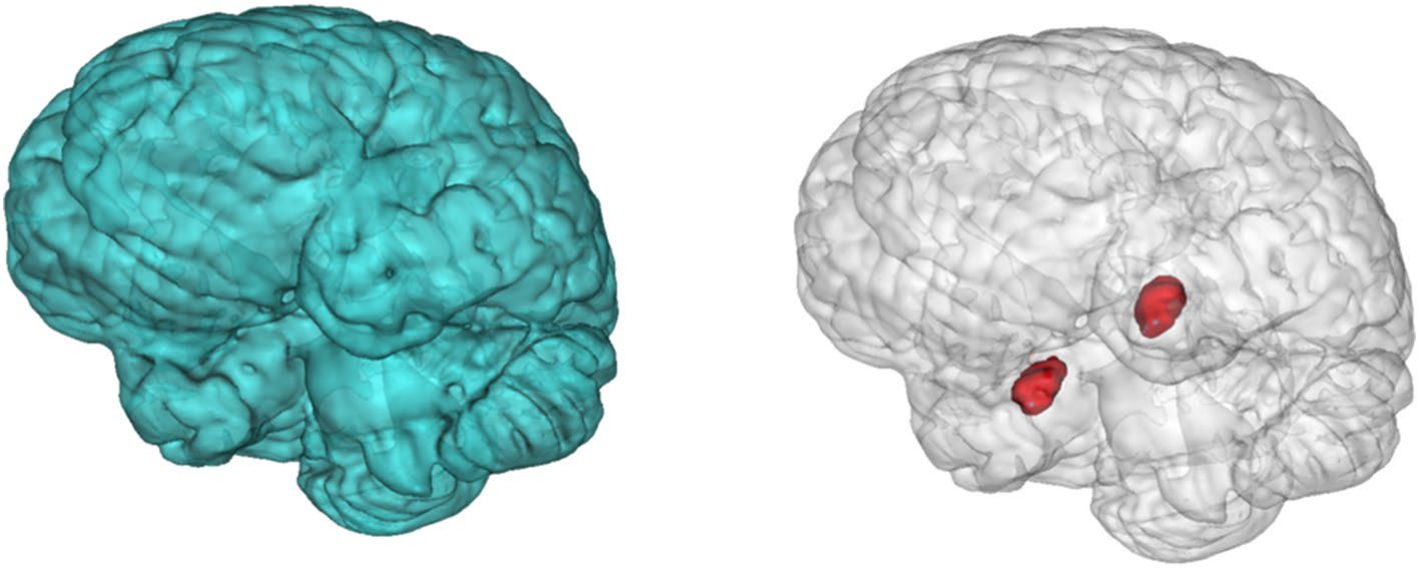
T1-weighted MRI scan showing the total brain (in blue) and amygdala (in red).

Next, we stratified the association by majority and minority statuses (Table 4). In children of Dutch majority group, ever being exposed to poverty was associated with smaller total brain (B =  − 0.21, 95%CI − 0.38; − 0.04, *p* = 0.02). The association was most obvious if exposure occurred in childhood (B =  − 0.23, 95%CI − 0.41; − 0.04, *p* = 0.02). Importantly, we found an association between exposure to childhood poverty and cerebral white matter volume in the majority population (B =  − 0.26, 95%CI − 0.45; − 0.06, *p* = 0.01, *p*_FDR-adjusted_ = 0.035) but not in the minority population (B =  − 0.05, 95%CI − 0.23; 0.12, *p* = 0.54, *p*_FDR-adjusted_ = 0.542); a significant interaction of ever being exposed to poverty with minority status was observed (*p* for interaction = 0.04) (Supplementary Table 8). Importantly, the association between poverty in childhood and cerebral white matter volume in the majority population, the Dutch origin children, were still significant after multiple comparison correction. Among minority children, ever being exposed to poverty in pregnancy was associated with smaller amygdala volume (B =  − 0.21, 95%CI − 0.37; − 0.05, *p* = 0.01, *p*_FDR-adjusted_ = 0.036). This trend towards smaller amygdala volume of children exposed to poverty in pregnancy was also observed in the majority children with the comparable effect size, but did not reach significance (B =  − 0.19, 95%CI − 0.40; 0.03, *p* = 0.09, *p*_FDR-adjusted_ = 0.208). However, only few majority children were exposed to poverty in pregnancy (majority: n = 57, minority: n = 245). No association with hippocampal volume was found in either group. The brain morphologies that differed by poverty status are shown in Fig. 3. This illustrates that the volume smaller in minority children exposed to poverty (i.e. amygdala volume; shown in red) is relatively small compared to the total brain volume associated with poverty exposure in majority children (shown in blue). As a follow-up analysis, child height, which was measured approximately 1–2 months prior to brain measurement, was added to the model to examine possible stunting as an indicator of general physical development. The associations between poverty and global brain volumes were slightly attenuated after adjusting for child height in the majority group, especially for the cortical gray matter volumes (total brain volume: B =  − 0.16, 95%CI − 0.33; 0.004, *p* = 0.06, cortical gray matter volume: B =  − 0.14, 95%CI − 0.31; 0.03, *p* = 0.12, *p*_FDR-adjusted_ = 0.232, cerebral white matter volume: B =  − 0.18, 95%CI − 0.35; − 0.01, *p* = 0.04, *p*_FDR-adjusted_ = 0.174).

**Table 4:** the association of poverty wth brain morphology among children of Dutch majority and non-Western minority ethnic groups (N = 1895).

	N	Total brain volume			Cortical gray matter volume			Cerebral white matter volume			Mean hippocampus volume			Mean amygdala volume		
		B	95%CI	*p* value	B	95%CI	*p* value	B	95%CI	*p* value	B	95%CI	*p* value	B	95%CI	*p* value
Dutch (N = 1365)																
Never poverty	1250	Ref.			Ref.			Ref.			Ref.			Ref.		
Ever poverty	115	− 0.21	− 0.38 to − 0.04	0.02	− 0.18	− 0.36 to − 0.004	0.04	− 0.22	− 0.40 to − 0.05	0.01	0.03	− 0.13 to 0.19	0.68	− 0.07	− 0.23 to 0.09	0.40
No poverty in pregnancy	1308	Ref.			Ref.			Ref.			Ref.			Ref.		
Poverty in pregnancy, all	57	− 0.17	− 0.41 to 0.06	0.15	− 0.18	− 0.42 to 0.06	0.14	− 0.17	− 0.41 to 0.07	0.16	0.09	− 0.13 to 0.31	0.41	− 0.19	− 0.40 to 0.03	0.09
No poverty in childhood	1272	Ref.			Ref.			Ref.			Ref.			Ref.		
Poverty in childhood, all	93	− 0.23	− 0.41 to − 0.04	0.02	− 0.18	− 0.37 to 0.01	0.07	− 0.26	− 0.45 to − 0.06	0.01 *	0.06	− 0.11 to 0.24	0.50	− 0.01	− 0.19 to 0.16	0.89
Non Western (N = 530)																
Never poverty	233	Ref.			Ref.			Ref.			Ref.			Ref.		
Ever poverty	297	− 0.03	− 0.20 to 0.15	0.76	− 0.07	− 0.24 to 0.11	0.45	0.01	− 0.17 to 0.19	0.90	− 0.12	− 0.28 to 0.03	0.13	− 0.15	− 0.31 to 0.004	0.06
No poverty in pregnancy	285	Ref.			Ref.			Ref.			Ref.			Ref.		
Poverty in pregnancy, all	245	− 0.02	− 0.19 to 0.16	0.86	− 0.06	− 0.24 to 0.11	0.48	0.04	− 0.14 to 0.22	0.67	− 0.10	− 0.26 to 0.06	0.21	− 0.21	− 0.37 to − 0.05	0.01 *
No poverty in childhood	310	Ref.			Ref.			Ref.			Ref.			Ref.		
Poverty in childhood, all	220	− 0.08	− 0.25 to 0.09	0.36	− 0.09	− 0.26 to 0.08	0.29	− 0.05	− 0.23 to 0.12	0.54	− 0.05	− 0.20 to 0.11	0.54	− 0.06	− 0.22 to 0.09	0.41

Models for Dutch adjusted for child age at brain measurement, child sex, maternal education at pregnancy, maternal IQ, maternal and paternal psychiatric symptoms at pregnancy.

Models for non-Western adjusted for Dutch model covariates + detailed maternal ethnicity [Cape Verdean, Moroccan, Dutch Antillean, Surinamese, Turkish, African, middle and south American and Asian (except for Indonesian and Japanese)].

Models for subcortical regions additionally adjusted for total intracranial volume.

All brain measures of outcome are standardized.

Ever poverty is a total of "poverty in pregnancy only", "poverty in childhood only" and "chronic poverty".

* indicates adjusted *p* values < 0.05.

Adjusted *p* values are obtained by considering the multiple comparisons (four tests for ever, pragnant any and childhood any poverty) with the Benjamini–Hochberg false discovery rate method.

### Poverty, child brain morphology, and school performance

**Figure 4:**
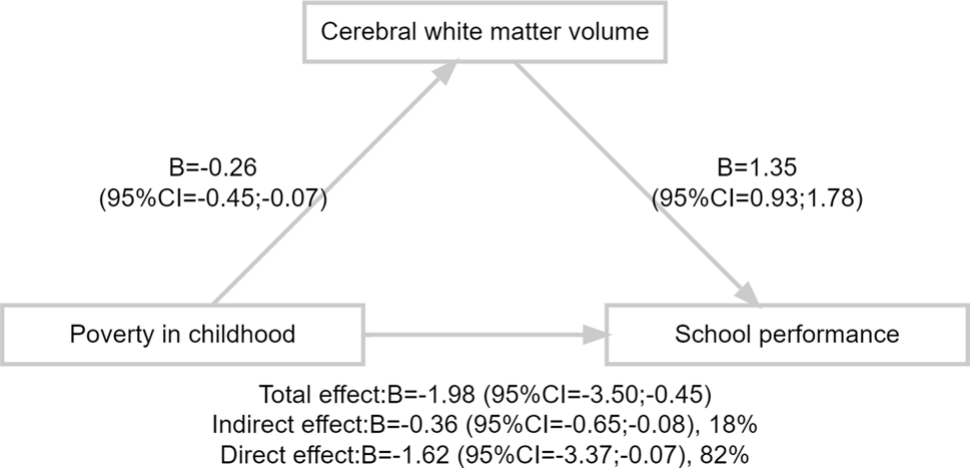
Mediating role of cerebral white matter volumes on the association between exposure to childhood poverty and school performance in children from Dutch majority group. Total sample: n = 1365. Model adjusted for: poverty → cerebral white matter volume: child age at brain measurement, child sex, maternal education at pregnancy, maternal IQ, maternal and paternal psychiatric symptoms at pregnancy; cerebral white matter volume → school performance: child age at CITO assessment, child age at brain measurement, child sex, maternal education at pregnancy, maternal IQ, maternal and paternal psychiatric symptoms at pregnancy; poverty → school performance: child age at CITO assessment, child sex, maternal education at pregnancy, maternal IQ, maternal and paternal psychiatric symptoms at pregnancy. CITO score was derived from a mandatory academic test conducted in the final grade of primary school and a proxy of school performance. Higher score indicates higher levels of school performance.

Next, we examined whether the association between being exposed to poverty in childhood and smaller cerebral white matter volumes in majority children underlies school performance. Child school performance were measured via the CITO test^36^, the most common mandatory academic examination conducted in primary school at a mean age of 12 years, which guides the choice for secondary education. In the current sample, CITO score was collected when children were approximately at age 11.9 (SD: 0.4). The test score was standardized, ranging from 500 to 550, with higher scores indicating better school performance. After we confirmed the association between being exposed to poverty in childhood and school performance (B =  − 1.98, 95%CI − 3.50; − 0.45, *p* = 0.01), and between cerebral white matter volume and school performance with multivariate linear regression (B = 1.35, 95%CI 0.93; 1.78, *p* < 0.01), causal mediation analysis was performed^37^. Difference in cerebral white matter volume explained the association between exposure to poverty in childhood and school performance as the indirect effect accounted for 18% of the total effect (indirect effect: B =  − 0.36, 95%CI − 0.65; − 0.08, *p* < 0.01) (Fig. 4). This demonstrates that smaller cerebral white matter volumes partially account for the association between living in poor household and less optimal school performance in Dutch majority children. We conducted the same analysis in the minority children, but found no association between being exposed to poverty in childhood and school performance (B = − 1.30, 95%CI − 2.93; 0.33, *p* = 0.12) as well as between being exposed to poverty in childhood and volumes of any brain areas, thus we did not perform a formal mediation analysis.

## Discussion

We found that overall exposure to poverty was not associated with child brain morphology at age 10 years. However, we found an association between ever being exposed to poverty in pregnancy and amygdala volume, which survived multiple testing, indicating the associations differed by timing of exposure. We also found evidence for a differential association by majority and minority groups. In particular, Dutch children exposed to poverty in childhood showed smaller cerebral white matter volumes than majority control. This association was not observed in minority population. Moreover, the association of being exposed to poverty in childhood with cerebral white matter volume underlay the differences in school performance only in Dutch majority children. These findings are an important addition to the literature for several reasons. We prospectively assessed poverty exposure from pregnancy onward and thus prior to brain assessment. This not only enabled us to infer temporal associations more reliably but to study the importance of timing of poverty experience. Further, our study comprised the largest sample outside of the US including participants of multiple national origins, which allowed us to assess differences between majority and minority groups. Importantly, we analyzed the association between poverty exposure and the preadolescent brain morphology also in relation to cognitive functions assessed after the neuroimaging.

Most studies report some associations between poverty and brain characteristics, but the evidence for an association with specific regional child brain morphology is mixed. A study assessing 1099 three-to-twenty-years-old people showed no cross-sectional association between income and volumes of total white matter, hippocampus, and amygdala^14^. In contrast, a longitudinal study found an association between lower income-to-need ratio and smaller cortical gray and white matter, hippocampus, and amygdala volumes^10^, similar to our results in the partially-adjusted models. These inconsistencies may lie in differences in target age and the confounders included. Also, the small effect size of poverty in the previous studies as well as in the current study suggest that brain development is largely determined by other social, lifestyle and genetic factors. Overall, we found no associations between ever-exposed to poverty and brain morphology, highlighting two additional explanations for the seemingly inconsistent findings that will be discussed below. First, we addressed the timing of exposure, while most childhood studies included a wide age range of poverty experience and did not distinguish between pregnancy and childhood poverty. Second, we stratified by majority/minority status considering the intersectionality and qualitative differences in poverty experience.

The current study is the first to prospectively examine differential associations of poverty experience with child brain morphology by developmental periods. We showed that the difference in amygdala volume related to low income was more pronounced if the exposure occurred in pregnancy, a critical brain developmental period^21^. During the prenatal period, the fetal brain undergoes the greatest growth including the neuronal migration and gyrification, and the total number of neurons for the lifetime is created^23^. In addition, the amygdala has a large number of cortisol receptors^12^, thus stress induced by poverty status in pregnancy may lead to a smaller amygdala volume as chronic stress causes hyperactivity, which after a prolonged period results in cellular atrophy and death^12^. Pregnant women in poverty may have limited access to material resources, social support and health care (including delayed pregnancy care) and are prone to risky behaviors including increased drug and alcohol consumption and unhealthy food intake^38^, all of which could lead to maternal stress in pregnancy or after birth. Maternal stress during pregnancy has repeatedly been related to systemic inflammation. Any such systemic maternal inflammatory process may trigger an inflammatory or immunological process in the fetal brain, leading to alterations in brain developmental processes. Animal studies suggest that this inflammatory or immunological process may impact axon growth, synapse formation and myelination^32,39,40^. Previous research has also shown some supporting findings: an association between prenatal stress, indexed by intrauterine concentration of cortisol ^32^ or interleukine-6^39^, and offspring amygdala volumetric differences; and an association of poverty exposure right after birth with lower total and subcortical gray matter volumes including amygdala in infancy^15^. This also could partially explain why we did not find associations of poverty with the global brain metrics, given that cortisol receptors are particularly prominent in the amygdala. However, in the absence of a biological stress measure, we cannot demonstrate that the association between poverty in pregnancy and smaller amygdala volume is explained by stress experienced in pregnancy.

Our study also revealed differences in the association by majority/minority status. Children from the Dutch origin majority who were exposed to poverty showed smaller total brain volumes. This association was not found in children from non-Western minority group, supporting heterogeneous associations between poverty and global brain morphology by majority and minority status. The smaller global brain volumes in children of Dutch majority group exposed to poverty might be indicative of cumulative exposure to neurodevelopmental burden due to socioeconomic disadvantage, poor diet, structural deprivation, and less familial reserves. Adjustment for child height, another indicator of global thriving, further provided the support for a stunting hypothesis, suggesting that the global brain differences in the Dutch group may partly reflect the effect of poverty on the global brain growth partly due to poor nutrition. The lack of association with global brain measures in non-Western minority children may suggest that minorities have familial or other resilience factors that reduce its impact on broader neurodevelopment^41,42^. We found that poverty experienced in pregnancy was associated with smaller amygdala volumes as a child. The non-Western children largely accounted for this association. The effect sizes in the children of Dutch origin were similar but non-significant given the small number of children exposed in utero. These results suggest a consistent association between intra-uterine exposure to poverty and smaller amygdala volume across majority and minority populations. We note that findings may not be easily generalizable to other populations if, as suggested by our results, the minority group status may interact with poverty.

The differences in global brain morphology in majority children mediated the association between poverty and later school performance, such that those exposed to childhood poverty had a lower CITO score (i.e. school performance) that could be accounted for by a cerebral white matter volume. This was in concordance with previous findings on the mediating role of volumes of frontal and temporal lobe on the association between poverty and child IQ^5^; likely, whole-brain surface area partially accounted for the association between household income and executive functions^14^. Our study adds to this evidence, suggesting that poverty during the first 5 years of life was associated with later child school performance through a potential impact on brain morphology. This may also shed some light on the intergenerational transmission of poverty via offspring brain development early in life as school performance is related to later socioeconomic success. Any causal interpretation of the mediation analysis, however, must keep in mind possible residual confounding (e.g. shared determinants of brain development and educational achievement) and possible biases in the assessment of educational achievement.

Our study had several limitations. First, a substantial number of participants did not undergo the imaging procedure. This decreased the power and introduced a bias, as people from lower socioeconomic backgrounds were more susceptible to loss to follow-up. Second, poverty status might be misclassified since income was self-reported. Previous studies revealed that more marginalized population more often declined to report their income and wages due to citizenship status, tax arrears or criminal justice involvement^43,44^. Although the official poverty prevalence in Rotterdam was similar to that observed in our population^34^, we have a sample selected towards higher socioeconomic status and more socially advantaged population. Thus, any generalization of the finding to other population needs to be conducted with some caution. Third, we measured brain morphology at one time point. Considering that brain developmental trajectories show an inverse U-shape^21^, we cannot confirm whether smaller volumes reflect delayed or accelerated development. However, given the age of our sample (9–11 years), most structures will not have started to decrease in volume yet. Also, the lack of a brain imaging assessment directly after birth and a relatively long time gap between income and brain measurement may be considered as a limitation. However, it is not clear what time interval is optimal to assess changes related to pregnancy and childhood exposure. Fourth, we could not use a continuous variable for family income to estimate the relationship with brain measurements. We did not assess income continuously but with several ordinal categories to reduce the non-response by participants that hesitate to specify their exact income. Fifth, we defined poverty as living under the low-income status and did not include measures indicative of a broader scope of poverty such as structural discrimination, poor access to health care and information, less social support and unhealthy behaviors due to stress. Therefore, we cannot attribute the brain volume differences to exposure to low income, but these are likely also indicative of non-monetary aspects of poverty.

We found that overall exposure to poverty was not associated with child brain morphology at age 10 years. However, we found an association between poverty in pregnancy and amygdala volume, indicating the associations differed by timing of exposure. In conclusion, our findings do not support an association between poverty ever experienced at any period in early-life and preadolescent brain morphology, but suggest that poverty exposure during pregnancy is associated with smaller amygdala in preadolescence. Additionally, we found that differential associations across majority and minority groups may exist, showing associations between childhood poverty and white matter volumes only in Dutch majority children. This suggested that majority group may be impacted more by the cumulative exposure to socioeconomic disadvantage. Further, smaller cerebral white matter volumes of majority children partly underlie less optimal school performance due to poverty. If replicated with repeated MRI assessments with larger sample size, our findings could provide scientific support for anti-poverty programs aimed to tackle different mechanisms and possibly distinct vulnerabilities by timing of exposure and across majority and minority groups.

## Methods

### Participants

Our study was embedded in the Generation R Study, a prospective population-based birth cohort in Rotterdam, the Netherlands. Pregnant women with an expected delivery date from April 2002 to January 2006 were invited. The study was described in detail elsewhere^33^, approved by the Medical Ethics Committee of the Erasmus Medical Center, and performed following the Declaration of Helsinki. Written informed consent was obtained from all adult participants.

In total, 5311 pregnant women provided data on standardized household income (i.e. data on household income and family size) in pregnancy. Of these, those without data on standardized household income in childhood (n = 110), and children without brain magnetic resonance imaging (MRI) data (n = 2413) were excluded. Further, 500 children were excluded due to: poor MRI data quality (n = 414), having braces (n = 58), different T1 acquisition (n = 19), or incidental findings (n = 9). Siblings were randomly excluded (n = 122) to keep only one child from each household. A total of 2166 children were included in our analytical sample (Fig. 1).

### Poverty

We defined poverty as living under the national low-income threshold in the Netherlands ^e.g.^^34^. Low-income threshold was set to the welfare benefit level of a one-person household in 1979, adjusted for purchasing power taking into account the price change over time^34^. An equivalence factor, which was determined based on the number of adults and children and the age of children of household, was used to make incomes of different types of households mutually comparable^45^. For example, the low-income threshold for single person was 9435 euros per year, while the threshold for household of married couple with two children was 15,543 euros and that for single parent with two children was 14,164 euros in the year 2000^34^. The number of adults and children living of the same income and the monthly disposable household income were reported at 30 weeks of pregnancy and twice during childhood, when children were 3 and 5 years old. The latter assessments were combined, as income stability is high during early childhood^5^. Missing values in family size were imputed using available data at other time points. Income data was originally collected in categories and recoded as numeric variables by taking the midpoint of each bin. The top category for each income assessment was filled with estimates obtained with the Pareto Curve^46^. The standardized household income was calculated from the family size and the household income. By comparing to the national low-income threshold, children’s poverty exposure was categorized as “never” or “ever” depending on whether their family experienced poverty at any assessment period. To assess the impact of specific time periods, we defined another category of poverty experience, as “poverty in pregnancy” versus “no poverty in pregnancy” and “poverty in childhood” versus “no poverty in childhood”. For the assessment of specific periodical impacts, the “ever poverty” exposure was further categorized as “poverty in pregnancy only”, “poverty in childhood only”, or “chronic poverty (poverty in both pregnancy and childhood)”.

### Brain imaging

Neuroimaging data were collected with structural acquisition and processing protocols, as described previously^47^. Brain MRI was conducted with a 3.0 Tesla MRI scanner (MR750w, General Electric, Milwaukee, WI, USA) using an 8-channel head coil. High-resolution T1-weighted structural MRI data were acquired with a 3D coronal inversion recovery fast spoiled gradient recalled sequence (repetition time = 8.77 ms, echo time = 3.4 ms, inversion time = 600 ms, flip angle = 10°, acquisition matrix = 220 × 220, field of view = 220 mm × 220 mm, slice thickness = 1.0 mm, number of slices = 230, ARC acceleration factor = 2). Details could be found elsewhere^47^. Data were processed using the FreeSurfer version 6.0 analysis suite^48^. Images were processed for cortical reconstruction and volumetric segmentation to obtain the volumes of regions of interests, i.e. total brain, cortical gray matter, cerebral white matter, hippocampus, and amygdala^49^. Data quality of the MRI scans was rated systematically by comparing the white and pial surface representations against the brain image at several slices, and brain scans deemed as unsuitable for analyses were excluded (Fig. 1)^47,49^. We compared children participating in the MRI assessment and those not included due to poor imaging quality data (Supplementary Table 1).

### Covariates

Maternal education, maternal and paternal psychiatric symptoms, and maternal ethnicity were assessed at pregnancy. Maternal education was categorized as “low” to “high” based on the Dutch standard classification of education ^50^ in accordance with the International Standard Classification of Education (ISCED)^51^. Psychiatric symptoms were evaluated using the Brief Symptom Inventory, a validated self-report questionnaire ^52,53^ and the Global Severity Index based on 53 items was used for analysis. Maternal ethnicity, which was defined by maternal national origin, was divided into “Dutch”, “Non-Dutch Western” and “Non-Western” based on the birthplace of the parents of the adult respondents, following the definitions used by the Statistics Netherlands ^35^ to define majority and minority statuses. Non-Dutch Western included European, American, Indonesian, Japanese and Oceanian. Non-Western included Cape Verdean, Moroccan, Dutch Antillean, Surinamese, Turkish, African, middle and south American and Asian (except for Indonesian and Japanese). Maternal intelligence quotient (IQ) was assessed when children were 5 to 7 years old as a non-verbal intelligence with a computerized version of the Ravens Advanced Progressive Matrices Test, set 1^54^. Child height was measured at the research center approximately 1–2 months prior to brain measurement using standardized procedures^55^.

School performance was measured with the CITO test, a mandatory academic test conducted in the final grade of primary school (children are on average 11 to 12 years old), most frequently used to guide the choice for secondary education. The test was developed by the Central Institute for Test Development (Centraal Instituut voor Test Ontwikkeling, CITO)^36^. Test score was standardized and ranged from 500 to 550, with higher score indicating higher levels of school performance.

### Non-response

There were some differences in socioeconomic status between children with complete data for poverty status and brain MRI (i.e. included sample) and those with no available data for income during childhood and brain MRI (i.e. excluded sample) (Supplementary Table 1). Briefly, children in poor households were less likely to participate in the follow-up assessments than children in nonpoor households. Also, childhood income and MRI data were more often available among higher educated mothers.

Missing covariate data (maximum missingness of 27.2% in paternal psychiatric symptoms) were imputed with multiple imputation by chained equations using predictive mean matching from the “mice” package ^56^ in R including exposure (household income) and outcomes (brain morphological measures) as well as covariates as predictors, and 30 imputed datasets were generated.

### Analyses

First, linear regression analyses were conducted to elucidate the association between exposure to poverty (never (reference) vs ever being exposed to poverty) and brain volumes (total brain, cortical gray matter, cerebral white matter, hippocampus and amygdala). Analyses were also performed by timing of exposure. Brain outcomes were standardized to allow comparison across metrics. We adjusted for child sex, child age at brain measurement, maternal ethnicity, maternal education, maternal IQ and maternal and paternal psychiatric symptoms. These variables were seen as potential confounders, hence included in the model. Intracranial volume was included in all models of hippocampus and amygdala volumes. In order to allow comparison of our results with those of previous research, we also present the partially-adjusted models. In model 1, child sex, child age at brain measurement, and maternal ethnicity were included as covariates. In a second model, we further adjusted for maternal education and maternal IQ. We present the coefficients of the covariates from the ever-poverty model (Supplementary Table 9 and 10), although any interpretation may be limited by the fact that the confounders were selected based on their associations with poverty and brain morphology rather than to estimate covariate associations. The interaction between poverty and sex was examined in analyses using the ever-exposed to poverty category to confirm the homogeneity of results across girls and boys.

The analysis of the association between poverty exposure (never vs ever; no poverty in pregnancy vs poverty in pregnancy; and no poverty in childhood versus poverty in childhood) and brain volumes was repeated in Dutch and non-Western groups to examine effect modification by majority and minority groups. A formal interaction test was also performed by the addition of a multiplicative term (poverty × ethnicity). We did not further analyze the non-Dutch Western group since too few were exposed to poverty to provide reliable estimates (total: n = 271; ever being poor: n = 30). Analyses in the non-Western group were additionally adjusted for detailed maternal ethnicity. Results for total population and each majority/minority group were corrected for multiple comparisons with the Benjamini–Hochberg false discovery rate procedure that adjusted the significance thereshold for the associations between poverty exposure and brain outcomes (volumes of cortical gray matter, cerebral white matter, hippocampus, and amygdala) ^57,58^, thus accounting for four hypotheses tested.

As a follow-up analysis, we tested whether bodily stunting could be an underlying mechanism of the associations between poverty (never vs ever) and global brain metrics (volumes of total brain, cortical gray matter, and cerebral white matter) in the Dutch majority group by additionally adjusting for the age-standardized child height.

We further conducted the mediation analysis to examine whether cerebral white matter volumes accounted for the association between being exposed to poverty in childhood and school performance in Dutch majority children. To perform mediation analysis, we imputed missing data including exposure, outcomes, and covariates of the mediation analysis model with expectation–maximization algorithm with R package “Amelia II”^59^, which enabled us to obtain 1 imputed dataset that provides precise estimates as multiple imputation does. Thus, mediation analysis was conducted on this 1 acquired dataset using R package “mediation” ^37^. Mediation model included the same covariates as the main analysis, i.e. child sex, child age at brain measurement, maternal national origin, maternal education, maternal IQ, and maternal and paternal psychiatric symptoms. In the outcome model, child age at CITO measurement was additionally adjusted. Averaged causal mediation effect, averaged direct effect, total effect, and proportion of mediated were calculated using the nonparametric bootstrap for variance estimation with 1000 simulations. All analyses were performed with R version 3.6.3 ^60^.

## Supplementary Information

Supplementary Tables.

## Data Availability

All relevant summary data supporting the current study are available within the article and the supplementary information files. An additional unpublished data can be provided by the corresponding author upon reasonable request. Due to ethical and legal restrictions, individual-level data cannot be made publicly available, and are available upon request to the data manager Claudia Kruithof (c.kruithof@erasmusmc.nl) and subject to the local rules and regulations.
