# Supplementary material for: Poverty from fetal life onward and child brain morphology
Source: Sci Rep. 2023 Jan 23;13:1295. doi: 10.1038/s41598-023-28120-2 (PMC9870876; doi:10.1038/s41598-023-28120-2)
Supplement: Supplementary file 1 — Supplementary Tables. [file 41598_2023_28120_MOESM1_ESM.docx]

**Table of contents of supplementary materials**

Supplementary Table 1. Sample characteristics by mutually exclusive categorization of timing of exposure

Supplementary Table 2. Sample characteristics differences by MRI data availability

Supplementary Table 3. Sample characteristics differences between participants in the analytical sample and those who were lost to follow-up

Supplementary Table 4. Sample characteristics by maternal ethnicity

Supplementary Table 5. The associations of poverty with global brain morphology from the partially-adjusted models

Supplementary Table 6. The associations of poverty with subcortical regional brain morphology from the partially-adjusted models

Supplementary Table 7. The associations between poverty and brain morphology with interaction for sex

Supplementary Table 8. The associations between poverty and brain morphology with interaction of maternal ethnicity

Supplementary Table 9. The associations of covariates with brain morphology from the ever-poverty model

Supplementary Table 10. The associations of covariates with brain morphology by maternal ethnicity from the ever-poverty models

**Supplementary Table 1. Sample characteristics by mutually exclusive categorization of timing of exposure**

|  | Never poverty N = 1724 (79.6%) | |  | Ever poverty N = 442 | | Low-income timing | | | | | |
| --- | --- | --- | --- | --- | --- | --- | --- | --- | --- | --- | --- |
| Characteristics |  |  |  |  |  | Poverty in pregnancy only N = 111 (5.1%) | | Poverty in childhood only N = 116 (5.4%) | | Chronic poverty N = 215 (9.9%) | |
| **Child sex** |  |  |  |  |  |  |  |  |  |  |  |
| Male, N, % | 843 | 48.9 |  | 215 | 48.6 | 51 | 45.9 | 57 | 49.1 | 107 | 49.8 |
| Female, N, % | 881 | 51.1 |  | 227 | 51.4 | 60 | 54.1 | 59 | 50.9 | 108 | 50.2 |
|  |  |  |  |  |  |  |  |  |  |  |  |
| **Child age at MRI measurement (years), mean, SD** | 10.1 | 0.6 |  | 10.2 | 0.6 | 10.2 | 0.6 | 10.2 | 0.6 | 10.1 | 0.5 |
|  |  |  |  |  |  |  |  |  |  |  |  |
| **Child school performance (CITO score), mean, SD** | 539.9 | 7.7 |  | 534.0 | 9.2 | 536.2 | 9.0 | 534.3 | 8.9 | 532.7 | 9.3 |
|  |  |  |  |  |  |  |  |  |  |  |  |
| **Maternal ethnicity** |  |  |  |  |  |  |  |  |  |  |  |
| Dutch, N, % | 1250 | 72.5 |  | 115 | 26.0 | 22 | 19.8 | 58 | 50.0 | 35 | 16.3 |
| Non-Dutch Western, N, % | 241 | 14.0 |  | 30 | 6.8 | 12 | 10.8 | 6 | 5.2 | 12 | 5.6 |
| Non-Western, N, % | 233 | 13.5 |  | 297 | 67.2 | 77 | 69.4 | 52 | 44.8 | 168 | 78.1 |
|  |  |  |  |  |  |  |  |  |  |  |  |
| **Maternal education at pregnancy ^a^** |  |  |  |  |  |  |  |  |  |  |  |
| High, N, % | 684 | 39.7 |  | 17 | 3.8 | 7 | 6.3 | 6 | 5.2 | 4 | 1.9 |
| Mid-high, N, % | 492 | 28.5 |  | 67 | 15.2 | 19 | 17.1 | 28 | 24.1 | 20 | 9.3 |
| Mid-low, N, % | 435 | 25.2 |  | 172 | 38.9 | 46 | 41.4 | 54 | 46.6 | 72 | 33.5 |
| Low, N, % | 113 | 6.6 |  | 186 | 42.1 | 39 | 35.1 | 28 | 24.1 | 119 | 55.3 |
|  |  |  |  |  |  |  |  |  |  |  |  |
| **Maternal IQ ^b^, mean, SD** | 101.0 | 12.5 |  | 90.3 | 15.0 | 95.0 | 14.1 | 93.9 | 15.0 | 86.0 | 14.4 |
|  |  |  |  |  |  |  |  |  |  |  |  |
| **Parental psychiatric symptoms at pregnancy** |  |  |  |  |  |  |  |  |  |  |  |
| Mother ^c^, median, IQR | 0.12 | 0.2 |  | 0.27 | 0.5 | 0.25 | 0.4 | 0.19 | 0.3 | 0.39 | 0.7 |
| Father ^d^, median, IQR | 0.06 | 0.1 |  | 0.12 | 0.2 | 0.10 | 0.2 | 0.06 | 0.2 | 0.17 | 0.3 |

The data was combined across imputed datasets.

Non-Dutch-Western includes Indonesian, American, Asian, European, Oceanian. Non-Western includes Cape Verdean, Moroccan, Dutch Antilles, Surinamese, Turkish, African, American non-Western, Asian non-Western.

Ever poverty is a total of "poverty in pregnancy only", "poverty in childhood only" and "chronic poverty".

a: missing data N = 54 (2.5%)

b: missing data N = 138 (6.4%)

c: missing data N = 233 (10.8%)

d: missing data N = 583 (26.9%)

**Supplementary Table 2. Sample characteristics differences by MRI data availability**

| Characteristics |  | A: With good quality brain MRI data (N = 2288) | | B: With poor quality brain MRI data (N = 500) | | C: Without MRI data (N = 2413) | | P-value  (A vs B) ^a^ | P-value  (A vs C) ^a^ |
| --- | --- | --- | --- | --- | --- | --- | --- | --- | --- |
| Income in pregnancy, N, % | Ever | 338 | 14.8 | 88 | 17.6 | 480 | 19.9 | 0.13 | < 0.01 |
| Income at 3yo, N, % | Ever | 213 | 11.6 | 65 | 16.2 | 276 | 11.4 | 0.02 | < 0.01 |
| Income at 5yo, N, % | Ever | 239 | 11.2 | 60 | 13.2 | 304 | 12.6 | 0.26 | < 0.01 |
| Child sex, N, % | Male | 1126 | 49.2 | 241 | 48.2 | 1235 | 51.2 | 0.72 | 0.19 |
| Child age at MRI measurement (years), mean, SD |  | 10.1 | 0.6 | 10.1 | 0.6 | 10.5 | 1.1 | 0.62 | <0.01 |
| Maternal national origins, N, % | Dutch | 1451 | 63.4 | 292 | 58.5 | 1435 | 59.5 | 0.07 | 0.01 |
|  | Non-Western | 548 | 24 | 143 | 28.7 | 676 | 28 |  |  |
| Maternal IQ, mean, SD |  | 99.5 | 13.7 | 98.1 | 14.7 | 97 | 14.6 | 0.06 | < 0.01 |
| Maternal education at pregnancy, N, % | High | 743 | 33.3 | 143 | 29.4 | 667 | 27.6 | 0.04 | < 0.01 |
|  | Mid-high | 580 | 26 | 112 | 23 | 530 | 22 |  |  |
|  | Mid-low | 619 | 27.7 | 149 | 30.7 | 683 | 28.3 |  |  |
| Parental psychiatric symptoms at pregnancy, median, IQR | Mother | 0.2 | 0.2 | 0.3 | 0.3 | 0.27 | 0.3 | 0.04 | < 0.01 |
|  | Father | 0.1 | 0.1 | 0.1 | 0.1 | 0.14 | 0.2 | 0.27 | 0.03 |
| Maternal age at child birth, mean, SD |  | 31.8 | 4.4 | 31.9 | 4.6 | 31.1 | 4.9 | 0.75 | < 0.01 |

a: P-values were derived from t-test for continuous variables and chi-square test for categorical variables.

**Supplementary Table 3. Sample characteristics differences between participants in the analytical sample and those who were lost to follow-up**

| Characteristics |  | With poverty data in pregnancy (N = 5311) | |  | With complete poverty and brain MRI data (N = 2788) | |  | With complete poverty and good quality brain MRI data (N = 2288) | |
| --- | --- | --- | --- | --- | --- | --- | --- | --- | --- |
| Income in pregnancy, N, % | Available | 5311 | 100.0 |  | 2788 | 100.0 |  | 2288 | 100.0 |
|  | Never | 4373 | 82.3 |  | 2362 | 84.7 |  | 1950 | 85.2 |
|  | Ever | 938 | 17.7 |  | 426 | 15.3 |  | 338 | 14.8 |
|  | Missing | 0 | 0.0 |  | 0 | 0.0 |  | 0 | 0.0 |
|  |  |  |  |  |  |  |  |  |  |
| Income at 3yo, N, % | Available | 3889 | 73.2 |  | 2239 | 80.3 |  | 1837 | 80.3 |
|  | Never | 3335 | 85.8 |  | 1961 | 87.6 |  | 1624 | 88.4 |
|  | Ever | 554 | 14.2 |  | 278 | 12.4 |  | 213 | 11.6 |
|  | Missing | 1422 | 26.8 |  | 549 | 19.7 |  | 451 | 19.7 |
|  |  |  |  |  |  |  |  |  |  |
| Income at 5yo, N, % | Available | 4652 | 87.6 |  | 2589 | 92.9 |  | 2134 | 93.3 |
|  | Never | 4049 | 87.0 |  | 2290 | 88.5 |  | 1895 | 88.8 |
|  | Ever | 603 | 13.0 |  | 299 | 11.5 |  | 239 | 11.2 |
|  | Missing | 659 | 12.4 |  | 199 | 7.1 |  | 154 | 6.7 |
|  |  |  |  |  |  |  |  |  |  |
| Child sex, N, % | Available | 5311 | 100.0 |  | 2788 | 100.0 |  | 2288 | 100.0 |
|  | Male | 2665 | 50.2 |  | 1367 | 49.0 |  | 1126 | 49.2 |
|  | Female | 2646 | 49.8 |  | 1421 | 51.0 |  | 1162 | 50.8 |
|  | Missing | 0 | 0.0 |  | 0 | 0.0 |  | 0 | 0.0 |
|  |  |  |  |  |  |  |  |  |  |
| Child age at MRI measurement (years), mean, SD |  | 10.1 | 0.6 |  | 10.1 | 0.6 |  | 10.1 | 0.6 |
|  | Missing | 2310 | 43.5 |  | 0 | 0.0 |  | 0 | 0.0 |
|  |  |  |  |  |  |  |  |  |  |
| Maternal ethnicity, N, % | Available | 5304 | 99.9 |  | 2787 | 100.0 |  | 2288 | 100.0 |
|  | Dutch | 3243 | 61.1 |  | 1743 | 62.5 |  | 1451 | 63.4 |
|  | Non-Dutch-Western | 655 | 12.3 |  | 353 | 12.7 |  | 289 | 12.6 |
|  | Non-Western | 1406 | 26.5 |  | 691 | 24.8 |  | 548 | 24.0 |
|  | Missing | 7 | 0.1 |  | 1 | 0.0 |  | 0 | 0.0 |
|  |  |  |  |  |  |  |  |  |  |
| Maternal IQ, mean, SD |  | 98.2 | 14.3 |  | 99.3 | 13.9 |  | 99.5 | 13.7 |
|  | Missing | 847 | 15.9 |  | 191 | 6.9 |  | 146 | 6.4 |
|  |  |  |  |  |  |  |  |  |  |
| Maternal education at pregnancy, N, % | Available | 5165 | 97.3 |  | 2720 | 97.6 |  | 2234 | 97.6 |
|  | High | 1572 | 30.4 |  | 886 | 32.6 |  | 743 | 33.3 |
|  | Mid-high | 1239 | 24.0 |  | 692 | 25.4 |  | 580 | 26.0 |
|  | Mid-low | 1481 | 28.7 |  | 768 | 28.2 |  | 619 | 27.7 |
|  | Low | 873 | 16.9 |  | 374 | 13.8 |  | 292 | 13.1 |
|  | Missing | 146 | 2.7 |  | 68 | 2.4 |  | 54 | 2.4 |
|  |  |  |  |  |  |  |  |  |  |
| Parental psychiatric symptoms at pregnancy, median, IQR | Mother | 0.15 | 0.3 |  | 0.14 | 0.2 |  | 0.14 | 0.2 |
|  | Missing | 696 | 13.1 |  | 301 | 10.8 |  | 252 | 11.0 |
|  | Father | 0.06 | 0.2 |  | 0.06 | 0.1 |  | 0.06 | 0.1 |
|  | Missing | 1575 | 29.7 |  | 784 | 28.1 |  | 619 | 27.1 |
|  |  |  |  |  |  |  |  |  |  |
| Maternal age at child birth, mean, SD |  | 31.5 | 4.7 |  | 31.9 | 4.5 |  | 31.8 | 4.4 |
|  | Missing | 0 | 0.0 |  | 0 | 0.0 |  | 0 | 0.0 |

**Supplementary Table 4. Sample characteristics by maternal ethnicity**

| Characteristics |  | Dutch N = 1365 | |  | Non-Western N = 530 | |
| --- | --- | --- | --- | --- | --- | --- |
| Child sex, N, % | Male | 679 | 49.7 |  | 254 | 47.9 |
|  | Female | 686 | 50.3 |  | 276 | 52.1 |
|  |  |  |  |  |  |  |
| Child age at MRI measurement (years), mean, SD |  | 10.1 | 0.6 |  | 10.1 | 0.6 |
|  |  |  |  |  |  |  |
| Maternal education at pregnancy, N, % | High | 533 | 39.0 |  | 59 | 11.1 |
|  | Mid-high | 401 | 29.4 |  | 79 | 14.9 |
|  | Mid-low | 328 | 24.0 |  | 215 | 40.6 |
|  | Low | 103 | 7.5 |  | 177 | 33.4 |
|  |  |  |  |  |  |  |
| Maternal IQ, mean, SD |  | 102.0 | 12.3 |  | 90.7 | 14.6 |
|  |  |  |  |  |  |  |
| Parental psychiatric symptoms at pregnancy, median, IQR | Mother | 0.12 | 0.2 |  | 0.23 | 0.5 |
|  | Father | 0.06 | 0.1 |  | 0.10 | 0.2 |
|  |  |  |  |  |  |  |
| Poverty experience, N, % | Never | 1250 | 91.6 |  | 233 | 44.0 |
|  | Ever | 115 | 8.4 |  | 297 | 56.0 |
|  | Pregnancy | 22 | 1.6 |  | 77 | 14.5 |
|  | Early childhood | 58 | 4.2 |  | 52 | 9.8 |
|  | Chronic | 35 | 2.6 |  | 168 | 31.7 |

The data was combined across imputed datasets.

Non-Western includes Cape Verdean, Moroccan, Dutch Antilles, Surinamese, Turkish, African, American non-Western, Asian non-Western.

**Supplementary Table 5. The associations of poverty with global brain morphology from the partially-adjusted models**

|  |  | **Total brain volume** | | |  | **Cortical gray matter volume** | | |  | **Cerebral white matter volume** | | |
| --- | --- | --- | --- | --- | --- | --- | --- | --- | --- | --- | --- | --- |
|  | **N** | **B** | **95%CI** | **P-value** |  | **B** | **95%CI** | **P-value** |  | **B** | **95%CI** | **P-value** |
| **Model 1** |  |  |  |  |  |  |  |  |  |  |  |  |
| *Never poverty* | 1724 | ref. |  |  |  | ref. |  |  |  | ref. |  |  |
| *Ever poverty* | 442 | -0.16 | -0.26 to -0.06 | < 0.01 |  | -0.18 | -0.28 to -0.08 | < 0.01 |  | -0.13 | -0.23 to -0.03 | 0.02 |
| Poverty in pregnancy only | 111 | -0.08 | -0.25 to 0.09 | 0.34 |  | -0.13 | -0.30 to 0.04 | 0.14 |  | -0.03 | -0.21 to 0.14 | 0.72 |
| Poverty in childhood only | 116 | -0.20 | -0.36 to -0.04 | 0.02 |  | -0.18 | -0.34 to -0.02 | 0.03 |  | -0.19 | -0.35 to -0.02 | 0.02 |
| Chronic poverty | 215 | -0.19 | -0.32 to -0.05 | 0.01 |  | -0.20 | -0.34 to -0.07 | < 0.01 |  | -0.14 | -0.28 to -0.01 | 0.04 |
| **Model 2** |  |  |  |  |  |  |  |  |  |  |  |  |
| *Never poverty* |  | ref. |  |  |  | ref. |  |  |  | ref. |  |  |
| *Ever poverty* |  | -0.10 | -0.21 to 0.01 | 0.06 |  | -0.11 | -0.22 to 0.004 | 0.06 |  | -0.09 | -0.21 to 0.02 | 0.10 |
| Poverty in pregnancy only |  | -0.03 | -0.20 to 0.14 | 0.76 |  | -0.07 | -0.25 to 0.11 | 0.46 |  | 0.00 | -0.17 to 0.18 | 0.97 |
| Poverty in childhood only |  | -0.14 | -0.30 to 0.02 | 0.09 |  | -0.12 | -0.28 to 0.05 | 0.16 |  | -0.16 | -0.32 to 0.01 | 0.07 |
| Chronic poverty |  | -0.12 | -0.26 to 0.03 | 0.11 |  | -0.12 | -0.27 to 0.02 | 0.10 |  | -0.10 | -0.25 to 0.05 | 0.17 |

Model 1 adjusted for child age at brain measurement, child sex and maternal ethnicity.

Model 2: model 1 + maternal IQ and maternal educational attainment at pregnancy.

All brain measures of outcome are standardized.

Ever poverty is a total of "poverty in pregnancy only", "poverty in childhood only" and "chronic poverty".

**Supplementary Table 6. The associations of poverty with subcortical regional brain morphology from the partially-adjusted models**

|  |  | **Mean hippocampus volume** | | |  | **Mean amygdala volume** | | |
| --- | --- | --- | --- | --- | --- | --- | --- | --- |
|  | **N** | **B** | **95%CI** | **P-value** |  | **B** | **95%CI** | **P-value** |
| **Model 1** |  |  |  |  |  |  |  |  |
| *Never poverty* | 1724 | ref. |  |  |  | ref. |  |  |
| *Ever poverty* | 442 | -0.03 | -0.12 to 0.07 | 0.61 |  | -0.07 | -0.16 to 0.03 | 0.17 |
| Poverty in pregnancy only | 111 | -0.10 | -0.26 to 0.06 | 0.22 |  | -0.14 | -0.30 to 0.02 | 0.09 |
| Poverty in childhood only | 116 | -0.06 | -0.21 to 0.09 | 0.43 |  | 0.05 | -0.10 to 0.20 | 0.48 |
| Chronic poverty | 215 | 0.04 | -0.09 to 0.17 | 0.52 |  | -0.11 | -0.23 to 0.02 | 0.09 |
| **Model 2** |  |  |  |  |  |  |  |  |
| *Never poverty* |  | ref. |  |  |  | ref. |  |  |
| *Ever poverty* |  | -0.03 | -0.13 to 0.08 | 0.60 |  | -0.10 | -0.20 to 0.002 | 0.05 |
| Poverty in pregnancy only |  | -0.10 | -0.26 to 0.06 | 0.22 |  | -0.17 | -0.33 to -0.01 | 0.04 |
| Poverty in childhood only |  | -0.06 | -0.21 to 0.09 | 0.44 |  | 0.03 | -0.13 to 0.18 | 0.74 |
| Chronic poverty |  | 0.05 | -0.09 to 0.18 | 0.49 |  | -0.16 | -0.29 to -0.02 | 0.02 |

Model 1 adjusted for child age at brain measurement, child sex and maternal ethnicity.

Model 2: model 1 + maternal IQ and maternal educational attainment at pregnancy.

All brain measures of outcome are standardized.

Ever poverty is a total of "poverty in pregnancy only", "poverty in childhood only" and "chronic poverty".

**Supplementary Table 7. The associations between poverty and brain morphology with interaction for sex**

|  | Interaction term | | |
| --- | --- | --- | --- |
|  | B | 95%CI | P-value |
| Total brain volume | 0.02 | -0.15 to 0.20 | 0.78 |
|  |  |  |  |
| Cerebral gray matter volume | -0.02 | -0.19 to 0.16 | 0.85 |
|  |  |  |  |
| Cerebral white matter volume | 0.04 | -0.14 to 0.21 | 0.70 |
|  |  |  |  |
| Mean hippocampus volume | 0.06 | -0.10 to 0.22 | 0.47 |
|  |  |  |  |
| Mean amygdala volume | -0.06 | -0.22 to 0.11 | 0.49 |

Models adjusted for child age at brain measurement, maternal ethnicity, maternal education at pregnancy, maternal IQ, maternal and paternal psychiatric symptoms at pregnancy.

Models for subcortical structures (i.e., hippocampus and amygdala volumes) further adjusted for total intracranial volume.

All brain measures of outcome are standardized.

Interaction term was made between poverty status (never vs ever) and child sex (boy vs girl).

**Supplementary Table 8. The associations between poverty and brain morphology with interaction of maternal ethnicity**

|  | Interaction term | | |
| --- | --- | --- | --- |
|  | B | 95%CI | P-value |
| Total brain volume | 0.22 | -0.001 to 0.43 | 0.05 |
|  |  |  |  |
| Cerebral gray matter volume | 0.17 | -0.05 to 0.39 | 0.14 |
|  |  |  |  |
| Cerebral white matter volume | 0.24 | 0.01 to 0.46 | 0.04 |
|  |  |  |  |
| Mean hippocampus volume | -0.13 | -0.34 to 0.07 | 0.20 |
|  |  |  |  |
| Mean amygdala volume | -0.07 | -0.28 to 0.13 | 0.48 |

Models adjusted for child age at brain measurement, child sex, maternal education at pregnancy, maternal IQ, maternal and paternal psychiatric symptoms at pregnancy.

Models for subcortical structures (i.e., hippocampus and amygdala volume) further adjusted for total intracranial volume.

All brain measures of outcome are standardized.

Interaction term was made between poverty status (never vs ever) and maternal ethnicity (Dutch vs non-Western).

**Supplementary Table 9. The associations of covariates with brain morphology from the ever-poverty model**

|  | **Total brain volume** | |  | **Cortical gray matter volume** | |  | **Cerebral white matter volume** | |  | **Mean hippocampus volume** | |  | **Mean amygdala volume** | |
| --- | --- | --- | --- | --- | --- | --- | --- | --- | --- | --- | --- | --- | --- | --- |
|  | **B** | **95%CI** |  | **B** | **95%CI** |  | **B** | **95%CI** |  | **B** | **95%CI** |  | **B** | **95%CI** |
| Child age at brain measurement | 0.07 | 0.01 to 0.13 |  | -0.04 | -0.10 to 0.03 |  | 0.17 | 0.11 to 0.23 |  | 0.05 | -0.005 to 0.11 |  | 0.00 | -0.05 to 0.06 |
| Child sex: girl | -1.03 | -1.10 to -0.96 |  | -0.96 | -1.03 to -0.89 |  | -0.96 | -1.03 to -0.88 |  | -0.09 | -0.17 to -0.02 |  | -0.24 | -0.32 to -0.16 |
| Maternal ethnicity: Non-Dutch Western | -0.01 | -0.12 to 0.10 |  | -0.05 | -0.16 to 0.06 |  | 0.04 | -0.07 to 0.16 |  | 0.03 | -0.11 to 0.16 |  | 0.00 | -0.10 to 0.11 |
| Maternal ethnicity: Non western | -0.34 | -0.44 to -0.24 |  | -0.39 | -0.49 to -0.28 |  | -0.23 | -0.33 to -0.12 |  | 0.04 | -0.06 to 0.14 |  | -0.05 | -0.15 to 0.05 |
| Maternal IQ | 0.00 | -0.003 to 0.003 |  | 0.00 | -0.003 to 0.003 |  | 0.00 | -0.004 to 0.002 |  | 0.00 | -0.002 to 0.004 |  | 0.00 | -0.002 to 0.003 |
| Maternal education: Low | -0.20 | -0.34 to -0.06 |  | -0.23 | -0.37 to -0.09 |  | -0.13 | -0.27 to 0.02 |  | 0.03 | -0.11 to 0.16 |  | 0.12 | -0.009 to 0.25 |
| Maternal education: Mid-low | -0.17 | -0.27 to -0.07 |  | -0.20 | -0.30 to -0.10 |  | -0.11 | -0.21 to -0.01 |  | 0.00 | -0.09 to 0.10 |  | 0.07 | -0.02 to 0.16 |
| Maternal education: Mid-high | -0.09 | -0.19 to 0.001 |  | -0.12 | -0.22 to -0.03 |  | -0.05 | -0.14 to 0.05 |  | 0.04 | -0.05 to 0.13 |  | 0.03 | -0.06 to 0.12 |
| Maternal psychiatry symptoms | 0.01 | -0.13 to 0.14 |  | 0.03 | -0.11 to 0.17 |  | 0.01 | -0.13 to 0.15 |  | 0.13 | 0.0004 to 0.27 |  | 0.05 | -0.08 to 0.17 |
| Paternal psychiatry symptoms | -0.12 | -0.32 to 0.07 |  | -0.09 | -0.29 to 0.10 |  | -0.16 | -0.36 to 0.05 |  | 0.03 | -0.16 to 0.22 |  | 0.01 | -0.18 to 0.19 |

All brain measures of outcome are standardized.

Ever poverty is a total of "poverty in pregnancy only", "poverty in childhood only" and "chronic poverty".

Models for subcortical structures (i.e., hippocampus and amygdala volume) further adjusted for total intracranial volume.

**Supplementary Table 10. The associations of covariates with brain morphology by maternal ethnicity from the ever-poverty models**

|  | **Total brain volume** | |  | **Cortical gray matter volume** | |  | **Cerebral white matter volume** | |  | **Mean hippocampus volume** | |  | **Mean amygdala volume** | |
| --- | --- | --- | --- | --- | --- | --- | --- | --- | --- | --- | --- | --- | --- | --- |
|  | **B** | **95%CI** |  | **B** | **95%CI** |  | **B** | **95%CI** |  | **B** | **95%CI** |  | **B** | **95%CI** |
| **Majority status** |  |  |  |  |  |  |  |  |  |  |  |  |  |  |
| Child age at brain measurement | 0.06 | -0.01 to 0.14 |  | -0.03 | -0.11 to 0.05 |  | 0.16 | 0.08 to 0.24 |  | 0.05 | -0.03 to 0.12 |  | 0.00 | -0.08 to 0.07 |
| Child sex: girl | -1.04 | -1.13 to -0.95 |  | -0.96 | -1.05 to -0.87 |  | -0.96 | -0.11 to -0.86 |  | -0.12 | -0.22 to -0.02 |  | -0.23 | -0.33 to -0.13 |
| Maternal IQ | 0.00 | -0.003 to 0.005 |  | 0.00 | -0.003 to 0.01 |  | 0.00 | -0.004 to 0.004 |  | 0.00 | -0.002 to 0.01 |  | 0.00 | -0.003 to 0.005 |
| Maternal education: low | -0.20 | -0.40 to -0.002 |  | -0.28 | -0.48 to -0.08 |  | -0.09 | -0.29 to 0.12 |  | -0.01 | -0.19 to 0.18 |  | 0.08 | -0.11 to 0.26 |
| Maternal education: mid-low | -0.08 | -0.20 to 0.05 |  | -0.11 | -0.23 to 0.02 |  | -0.01 | -0.14 to 0.12 |  | 0.02 | -0.09 to 0.14 |  | 0.09 | -0.03 to 0.20 |
| Maternal education: mid-high | -0.05 | -0.16 to 0.07 |  | -0.09 | -0.21 to 0.03 |  | 0.00 | -0.12 to 0.12 |  | 0.06 | -0.05 to 0.16 |  | 0.06 | -0.04 to 0.17 |
| Maternal psychiatry symptoms | -0.04 | -0.28 to 0.20 |  | -0.03 | -0.28 to 0.21 |  | -0.04 | -0.28 to 0.21 |  | 0.22 | -0.01 to 0.45 |  | 0.07 | -0.15 to 0.29 |
| Paternal psychiatry symptoms | -0.25 | -0.54 to 0.04 |  | -0.20 | -0.50 to 0.09 |  | -0.31 | -0.61 to -0.01 |  | -0.01 | -0.29 to 0.28 |  | 0.02 | -0.26 to 0.30 |
|  |  |  |  |  |  |  |  |  |  |  |  |  |  |  |
| **Minority status** |  |  |  |  |  |  |  |  |  |  |  |  |  |  |
| Child age at brain measurement | 0.08 | -0.05 to 0.21 |  | -0.08 | -0.21 to 0.05 |  | 0.21 | 0.08 to 0.34 |  | 0.01 | -0.11 to 0.13 |  | 0.03 | -0.09 to 0.14 |
| Child sex: girl | -1.07 | -1.22 to -0.93 |  | -1.04 | -1.19 to -0.89 |  | -0.96 | -1.11 to -0.81 |  | -0.11 | -0.26 to 0.05 |  | -0.30 | -0.45 to -0.14 |
| Maternal IQ | 0.00 | -0.01 to 0.01 |  | 0.00 | -0.01 to 0.005 |  | 0.00 | -0.01 to 0.01 |  | 0.00 | -0.01 to 0.005 |  | 0.00 | -0.003 to 0.008 |
| Maternal education: low | -0.30 | -0.61 to 0.01 |  | -0.30 | -0.61 to 0.01 |  | -0.26 | -0.58 to 0.05 |  | 0.08 | -0.20 to 0.35 |  | 0.24 | -0.03 to 0.52 |
| Maternal education: mid-low | -0.32 | -0.60 to -0.05 |  | -0.39 | -0.67 to -0.12 |  | -0.24 | -0.52 to 0.04 |  | 0.02 | -0.22 to 0.27 |  | 0.05 | -0.20 to 0.29 |
| Maternal education: mid-high | -0.19 | -0.48 to 0.11 |  | -0.18 | -0.48 to 0.11 |  | -0.14 | -0.45 to 0.17 |  | 0.05 | -0.21 to 0.32 |  | -0.07 | -0.34 to 0.20 |
| Maternal psychiatry symptoms | -0.01 | -0.19 to 0.18 |  | 0.02 | -0.17 to 0.21 |  | -0.02 | -0.21 to 0.18 |  | 0.12 | -0.05 to 0.29 |  | 0.09 | -0.08 to 0.26 |
| Paternal psychiatry symptoms | 0.06 | -0.25 to 0.38 |  | 0.11 | -0.21 to 0.42 |  | 0.03 | -0.31 to 0.37 |  | 0.10 | -0.20 to 0.40 |  | -0.02 | -0.33 to 0.29 |

All brain measures of outcome are standardized.

Models for hippocampus and amygdala were additionally adjusted for the total intracranial volume.
